# The landscape of cancer-associated transcript fusions in adult brain tumors: a longitudinal assessment in 140 patients with cerebral gliomas and brain metastases

**DOI:** 10.3389/fonc.2024.1382394

**Published:** 2024-07-17

**Authors:** Philippe Metellus, Clara Camilla, Emilie Bialecki, Nathalie Beaufils, Christine Vellutini, Eric Pellegrino, Pascale Tomasini, Manmeet S. Ahluwalia, Alireza Mansouri, Isabelle Nanni, L’Houcine Ouafik

**Affiliations:** ^1^ Aix Marseille Univ, Centre national de Recherche Scientifique (CNRS), INP, Inst Neurophysiopathol, Marseille, France; ^2^ Ramsay Santé, Hôpital Privé Clairval, Département de Neurochirurgie, Marseille, France; ^3^ Aix Marseille Univ, APHM, CHU Timone, Service d’OncoBiologie, Marseille, France; ^4^ Aix Marseille Univ, APHM, Oncologie multidisciplinaire et innovations thérapeutiques, Marseille, France; ^5^ Aix-Marseille Univ, Centre national de Recherche Scientifique (CNRS), Inserm, CRCM, Marseille, France; ^6^ Miami Cancer Institute, Baptist Health South Florida, Miami, FL, United States; ^7^ Herbert Wertheim College of Medicine, Florida International University, Miami, FL, United States; ^8^ Department of Neurosurgery, Penn State Cancer Institute, Hershey, PA, United States

**Keywords:** NTRK gene fusion, TMPRSS gene fusion, FGFR3 gene fusion, glioma, brain metastases, NGS analysis

## Abstract

**Background:**

Oncogenic fusions of neurotrophic receptor tyrosine kinase *NTRK1*, *NTRK2*, or *NTRK3* genes have been found in different types of solid tumors. The treatment of patients with TRK fusion cancer with a first-generation TRK inhibitor (such as larotrectinib or entrectinib) is associated with high response rates (>75%), regardless of tumor histology and presence of metastases. Due to the efficacy of TRK inhibitor therapy of larotrectinib and entrectinib, it is clinically important to identify patients accurately and efficiently with TRK fusion cancer. In this retrospective study, we provide unique data on the incidence of oncogenic *NTRK* gene fusions in patients with brain metastases (BM) and gliomas.

**Methods:**

140 samples fixed and paraffin-embedded tissue (FFPE) of adult patients (59 of gliomas [17 of WHO grade II, 20 of WHO grade III and 22 glioblastomas] and 81 of brain metastasis (BM) of different primary tumors) are analyzed. Identification of *NTRK* gene fusions is performed using next-generation sequencing (NGS) technology using Focus RNA assay kit (Thermo Fisher Scientific).

**Results:**

We identified an *ETV6 (5)::NTRK3* (*15*) fusion event using targeted next-generation sequencing (NGS) in one of 59 glioma patient with oligodendroglioma–grade II, IDH-mutated and 1p19q co-deleted at incidence of 1.69%. Five additional patients harboring *TMPRSS (2)::ERG (4)* were identified in pancreatic carcinoma brain metastasis (BM), prostatic carcinoma BM, endometrium BM and oligodendroglioma (grade II), IDH-mutated and 1p19q co-deleted. A *FGFR3 (17)::TACC3 (11)* fusion was identified in one carcinoma breast BM. Aberrant splicing to produce *EGFR* exons 2-7 skipping mRNA, and *MET* exon 14 skipping mRNA were identified in glioblastoma and pancreas carcinoma BM, respectively.

**Conclusions:**

This study provides data on the incidence of *NTRK* gene fusions in brain tumors, which could strongly support the relevance of innovative clinical trials with specific targeted therapies (larotrectinib, entrectinib) in this population of patients. *FGFR3 (17)::TACC3 (11)* rearrangement was detected in breast carcinoma BM with the possibility of using some specific targeted therapies and *TMPRSS (2)::ERG (4)* rearrangements occur in a subset of patients with, prostatic carcinoma BM, endometrium BM, and oligodendroglioma (grade II), IDH-mutated and 1p19q co-deleted, where there are yet no approved ERG-directed therapies.

## 1 Introduction

The tropomyosin receptor kinase (TRK) family of receptors is composed of TRKA, TRKB, and TRKC, which are neurotrophic tyrosine receptor kinase (NTRK) proteins encoded by the *NTRK1*, *NTRK2* and *NTRK3* genes, respectively (1, 2). These receptors are highly expressed in neural tissue and participate in the development and proper functioning of the central nervous system (CNS). These receptors activate the RAS/MAPK pathway and can also signal via the PI3K/AKT/mTOR and the PLCγ/PLK pathways, depending on which docking protein binds to the kinase domain. Via these pathways, the signal transduction leads to neuronal survival, development, proliferation, synaptic plasticity, neuronal differentiation as well as memory and cognition (3–5). These receptors and their signaling cascade are also implicated in neoplastic cells (6).

Although mutations and alternative splicing occur, fusions are the most common aberrations of NTRK in tumors. Gene fusion events that involve *NTRK* genes (*NTRK1*, *NTRK2* and *NTRK3*) occur when the 3’ region of a *NTRK* gene encoding the tyrosine kinase domain is joined in-frame with 5’-end of a fusion partner gene, either by intra- or inter-chromosomal rearrangement (7, 8). The resulting fusion oncogene leads to the production of chimeric protein, with a constitutive activation of the kinase, due to loss of the extracellular domain, continuous downstream signaling and thus proliferation and cell survival (7).

Among adult and pediatric patients, the incidence of *NTRK* gene fusions varies between <5% in solid cancers (e.g., lung cancer, colorectal cancer, glioma) and >75% in rare cancers (e.g., infantile fibrosarcoma, secretory breast, secretory carcinoma of the salivary gland) (9). The presence of *NTRK* gene fusions in both adult and pediatric populations suggests it may be one of the first oncogenic drivers that are both tissue- and age-agnostic (10–12).

Regarding the central nervous system (CNS) tumors, *NTRK* fusions occur in up to 2% of gliomas in adults (13, 14). The incidence in pediatric high-grade gliomas (HGG) and diffuse infiltrating pontine glioma is around 5% and even 40% in infants with non-brainstem HGG (15–18). *NTRK2* is the most common fusion partner of the NTRK family in pediatric brain tumors (7, 8). Nevertheless, the occurrence of *NTRK* gene fusions in different types of brain tumors in adults remain quite unexplored.

The *FGFR* family exists of four transmembrane tyrosine kinase receptors (FGFR 1-4). The FGFR plays an important role in embryonal CNS development and in tumorigenesis, regulating angiogenesis, proliferation, differentiation, migration, and survival. *FGFR* genomic alterations (amplification, mutations, and fusions) occur in ~ 8% of gliomas, with most aberrations occurring in *FGFR1* and *FGFR3* (19). Chromosomal translocations that fuse the tyrosine kinase domains of *FGFR1* or *FGFR3* and *TACC1* or *TACC3* have been identified in 2% to 4% of gliomas (20–22). The IDH1/2 wild-type (3.5%) but none of IDH1/2-mutant grade II and III gliomas harbored *FGFR3*-*TACC3* fusions (21). *FGFR*-*TACC* rearrangements are reported in 2.9% of glioblastoma (GBM) (21). These *FGFR* fusion genes, such as *FGFR3*-*TACC3*, are capable of ligand-independent dimerization by virtue of the newly fused coiled-coil domain and have demonstrated oncogenic potential *in vitro* and *in vivo* (20). Further, *FGFR3*-*TACC3* fusion has been reported as predictive of response to FGFR tyrosine kinase inhibitors both preclinically (20, 21) and clinically in various solid tumors including gliomas (21). Clinical trials with FGFR inhibitors in brain tumors are being conducted (23, 24). FGFR fusions also have been identified as oncogenic drivers in breast tumors, lung cancer, and bladder carcinomas. *FGFR3*-*TACC3* fusions were identified in a subset of bladder carcinomas (25), raising an interest in FGFR pathway inhibitors (23).

The detection of *NTRK* and *FGFR* gene fusions in brain tumors and brain metastases is relevant in clinical practice because these alterations can be predictive biomarkers and therapeutic targets for specific kinase inhibitors. *NTRK* and *FGFR* gene fusions result in overexpression of the fusion kinase and its constitutive activation, promoting tumor growth as a driver mutation. Both NTRK and FGFR inhibitors are currently being or already tested in clinical trials with patients who have fusions and available for treatment in tumors, respectively. Therefore, assessing *NTRK* and *FGFR* gene fusions in brain tumors and brain metastases could help identify those patients who could receive a clinically relevant response. Clinical outcomes from administering the above-targeted kinase inhibitors for patients could benefit from therapy if the *NTRK* and *FGFR* gene fusions were detected.

The incidence of cancer-associated fusions in patients with brain tumors remains poorly documented in France. Although several diagnostic approaches can be used to detect gene fusions, RNA-based next generation sequencing remains one of the most sensitive methods, as it can directly detect the transcribed product of gene fusion at the mRNA level (26). In this retrospective study, we herein report the results of detection of incidence of cancer-associated fusions in different types of brain tumors (gliomas and brain metastases).

## 2 Materials and methods

### 2.1 Patients’ selection

Patients were selected for the analysis of brain tumors samples to NGS. This retrospective, single-center, exploratory study was reviewed and approved by the Institutional Ethic Committee of Ramsay Santé. Patient samples were identified from the AP-HM tumor library (AC-2013-1786) using the electronic patient record.

The selection criteria were adult patient with histologically proven glioma and brain metastasis (BM) operated in Clairval hospital since February 2015, paraffin-embedded tissue samples (< 5 years old) available in the AP-HM biobank with clinico-radiological data. Written informed consent was obtained from all patients to use of their tumor samples for research purposes and no-opposition was obtained from all patients to use their personal and medical data.

Samples from 140 patients with a brain tumor (59 glioma samples including [17 glioma-grade II, 20 glioma-grade III and 22 glioma-grade IV] and 81 BM samples) were selected for this study.

Data collected included: sex of patients, age at time of surgery, patient habits (smoking), location of brain tumor, tumor histology with molecular data, origin of primary tumor for BM samples, previous treatments (chemotherapy, immunotherapy, radiotherapy, others, …), tumor percentage of samples analyzed, NGS results (transcript fusions), date of recurrence/progression, postoperative treatments, date of death and patient consent for scientific research.

### 2.2 RNA based Next-Generation Sequencing (NGS)

#### 2.2.1 Tumor specimens and RNA preparation

Blocks of FFPE tumor were obtained from the pathology archives of Clairval hospital (Marseille). Tumor-rich areas (80% to 90%) were dissected from unstained sections by comparison with a hematoxylin and eosin-stained slide (HES-slide). Total RNAs were extracted using the Maxwell^®^ RSC RNA FFPE Kit (Promega, Lyon, France) with treatment with DNAse. RNAs were eluted with 50 µL of elution buffer, and purified RNA was quantified with a Qubit fluorometer (Quantifluor RNA system, Promega).

#### 2.2.2 Detection of Gene Fusions

The panel RNA Oncomine™ FOCUS Assay (OFA) contains a targeted, multi-biomarker panel that enables highly sensitive and robust detection of known fusions covering > 284 isoforms from 23 fusion drivers associated with solid tumors (Solid Tumor Fusion Transcript Panel Oncomine Focus Assay (A35956) Thermo Fisher Scientific). The panel also includes control amplicons representing five housekeeping gene transcripts (MYC, ITGB7, HMBS, LRP1, TBP), as well as amplicons that detect exons in the 5’ and 3’ regions of 4 of the target kinases (ALK, RET, ROS, NTRK). The latter are used to evaluate whether relative overexpression of the 3’ kinase domain is indicative of a gene fusion.

Amplicons sequencing libraries were prepared with 10 ng of RNA, according to manufacturer’s instructions with Oncomine Focus RNA assay kit using the AmpliSeq Kit (Thermo Fisher Scientific). Briefly, reverse transcription of total RNA with the superscript IV VILO Master-mix (Thermo Fisher Scientific) and amplification with the multiplexed fusion pool. Library preparation was performed according to the manufacturer’s instructions and concentration was determined using a quantitative PCR with the Ion library TaqMan® Quantitation kit (cat. No. 44688002). Libraries typically have yields of 100-500 pM and below 100 pM the library is excluded. Final libraries were diluted, pooled and further processed on Ion spheres using Ion 530 Chef Kits (Thermo Fisher Scientific) on the Ion Chef (Thermo Fisher Scientific). Sequencing was performed on the Ion S5-XL System, with 500 flows, and subsequent quality assessment for the run was completed using the Torrent Suite Software v5.16 (Thermo Fisher Scientific). Obligatory run metrics were composed of mean RNA read length (> 60bp), mean raw accuracy (> 99%), and total sequencing reads (> x 21,000,000). Further sample-specific quality assessment and analysis was completed using Ion reporter software (v5.12 February 2020, Thermo Fisher Scientific). For RNA variant annotation, the « Oncomine focus-520-w2.5-Fusions-Single Sample” workflow was used with default parameters. The validation criteria for RNA samples were as follows: each sample must generate at least 50,000 reads mapped and have a minimum mean read length of 60 bp. Additionally, at least three of the five RNA internal controls (TBP, LRP1, ITGB7, MYC, and HMBS) must be called. Finally, RNA alterations were reported only if a minimum number of reads was reached: 20 for targeted fusions, 250 for non-targeted fusions, and 120 for exon skipping.

To evaluate the limit of detection of the Oncomine assay, a dilution (1/10) of Seraseq Fusion RNA reference materials (SeraSeq fusion RNA Mix v4; Part Code 0710-0497; Seracare Life sciences Inc., Milford, MA) was made in a background of GM24386 RNA (mild-type material). Seventeen clinically relevant RNA fusions, which include *TPM3*-*NTRK1*, *FGFR3-TACC3, ETV6*-*NTRK3*, *TFG*-*NTRK1*, *CCDC6-RET*, *CD74-ROS1*, *FGFR3-BAIAP2L1*, *KIF5B*-*RET*, *EML4*-*ALK*, *LMNA-NTRK1, NCOA4-RET*, *PAX8*-*PPARG1*, *SLC34A2*-*ROS1*, *SLC45A3*-*BRAF*, *TMPRSS2-ERG*, *EGFR variant III* and *MET ex 14* skipping, were tested using Seraseq Fusion RNA Reference Materials (Seracare Life Sciences Inc.), to evaluate analytical sensitivity of the assay.

#### 2.2.3 Mutation calling

For detection of gene fusions and gene expression, the raw data in FASTQ format generated by the S5-XL were aligned to a custom reference genome using Torrent Suite version 5.18, with alignment performed by TMAP (https://github.com/iontorrent/TMAP; accessed February 2021), following removal of adapter sequences by Cutadapt software version 1.2.1 (27). The custom reference genome was assembled to include sequences of the designed fusion transcripts, normal transcripts of the genes involved in the fusions, gene regions for differential expression analysis, and the entire hg19 reference genome. Quality control of the raw FASTQ data was performed internally by the Torrent Suite software. Additionally, sequence reads were manually inspected in the Integrative Genomics Viewer (IGV; Broad Institute, Cambridge, MA). Notably, variant calling was performed using Ion Reporter software. Subsequently, fusion partners identified by the amplicon panel were further confirmed by RT-PCR analysis.

### 2.3 RT-PCR validation of fusion transcripts

RT-PCR of *ETV6*-*NTRK3*, *TMPRSS2*-*ERG, FGFR3*-*TACC3* fusion transcripts in formalin-fixed paraffin-embedded sections was based on previously described methods (28). Briefly, RNA was isolated from two 30 μm paraffin sections and reverse-transcribed to cDNA as described by the manufacturer (Thermo Fisher Scientific). The cDNA was then subjected to PCR for *ETV6*-*NTRK3* using sense TEL971 (5’-ACCACATCATGGTCTCTCTGTCTCCC-3’) and antisense TRKC 1059 (5’- CAGTTCTCGCTTCAGCACGATG-3’) primers (24); for *TMPRSS2-ERG* using *TMPRSS2* sense primer (nt: 81-98; 5’-GAGGTGAAAGCGGGTGTG-3’) and *ERG* antisense primer (nt: 253-234; 5’-GGCACACTCAAACAACGACT-3’); for *FGFR3-TACC3* using *FGFR3* sense primer (nt:2514-2533; 5’-GACCTGGACCGTGTCCTTAC-3’) and *TACC3* antisense primer (nt: 2087-2067; 5’-TCTCCTCCTGTGTCGCCTTT-3’). PCR conditions were as follows: 94°C for 5 min, followed by 35 cycles of 94°C for 45 s, 60°C for 1 min, 72°C for 1 min and a final extension of 72°C for 10 min. The reaction produced a 110 bp, 180 bp, and 108 bp PCR fragments for *ETV6*-*NTRK3*, *TMPRSS2*-*ERG*, and *FGFR3-TACC3*; respectively. Amplified products were visualized by electrophoresis using 2% polyacrylamide gels stained with ethidium bromide. *ETV6*-*NTRK3*, *TMPRSS2-ERG*, *FGFR3-TACC3* amplification products were confirmed by sequencing of the PCR products.

### 2.4 Statistical analyses

The inclusion population was described according to all patient characteristics. A descriptive analysis of the variables of interest included the frequencies of patient demographics, histology of brain tumors, origin of primary tumors, transcript fusions and exons skipping genes, was carried out by SAS^®^ Software.

## 3 Results

### 3.1 Characteristics of patients and samples

**Figure 1:**
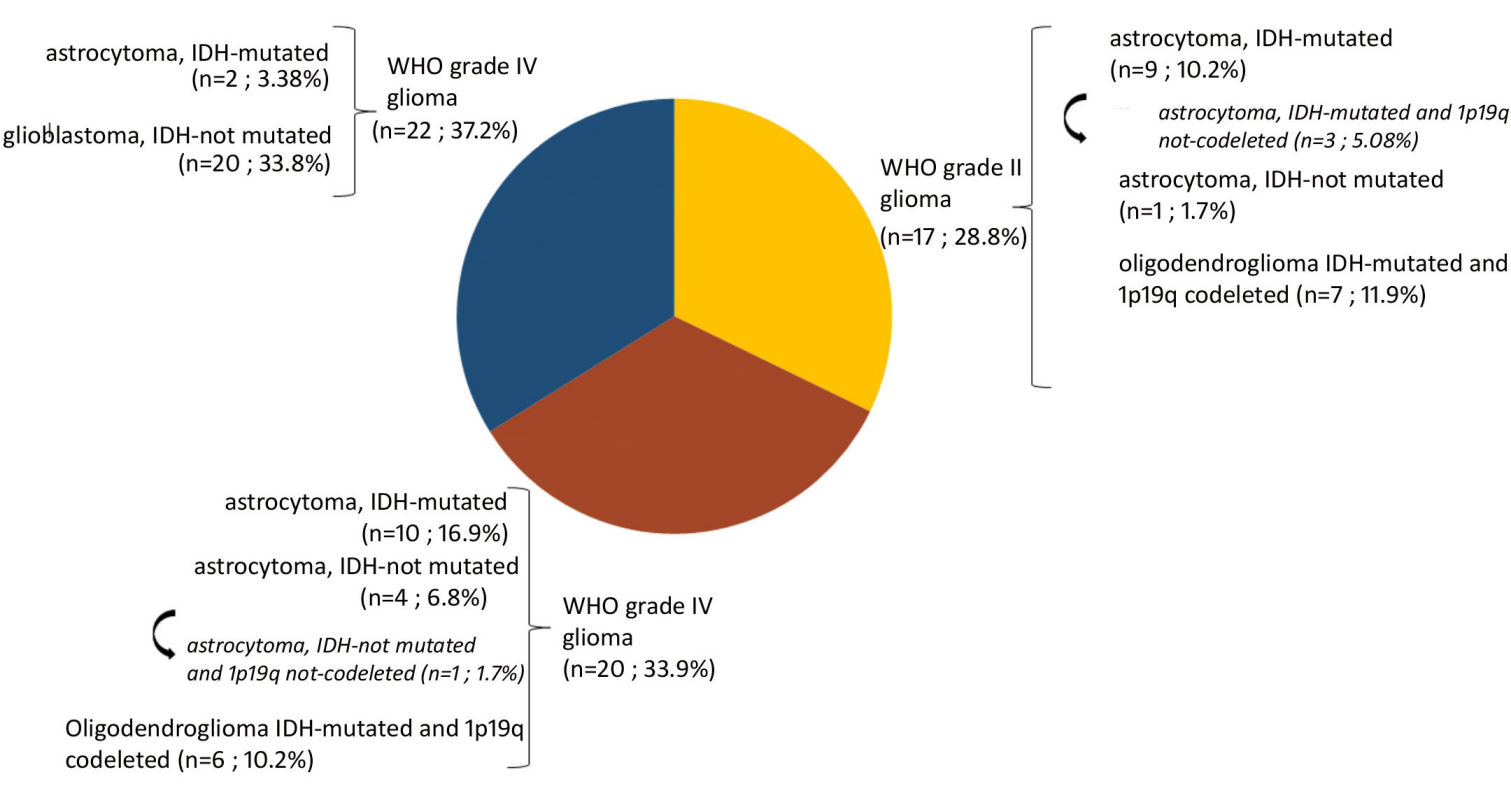
Prevalence of molecular profiles of gliomas samples analyzed. Astrocytoma IDH-mutated (n = 9; 10.2%) with three tumors (n = 3; 5.08%) do not present 1p19q co-deletion (arrow). Astrocytoma IDH-not mutated (n = 4; 6.8%) among which one tumor having 1p19q not co-deleted (1.7%) (arrow).

**Figure 2:**
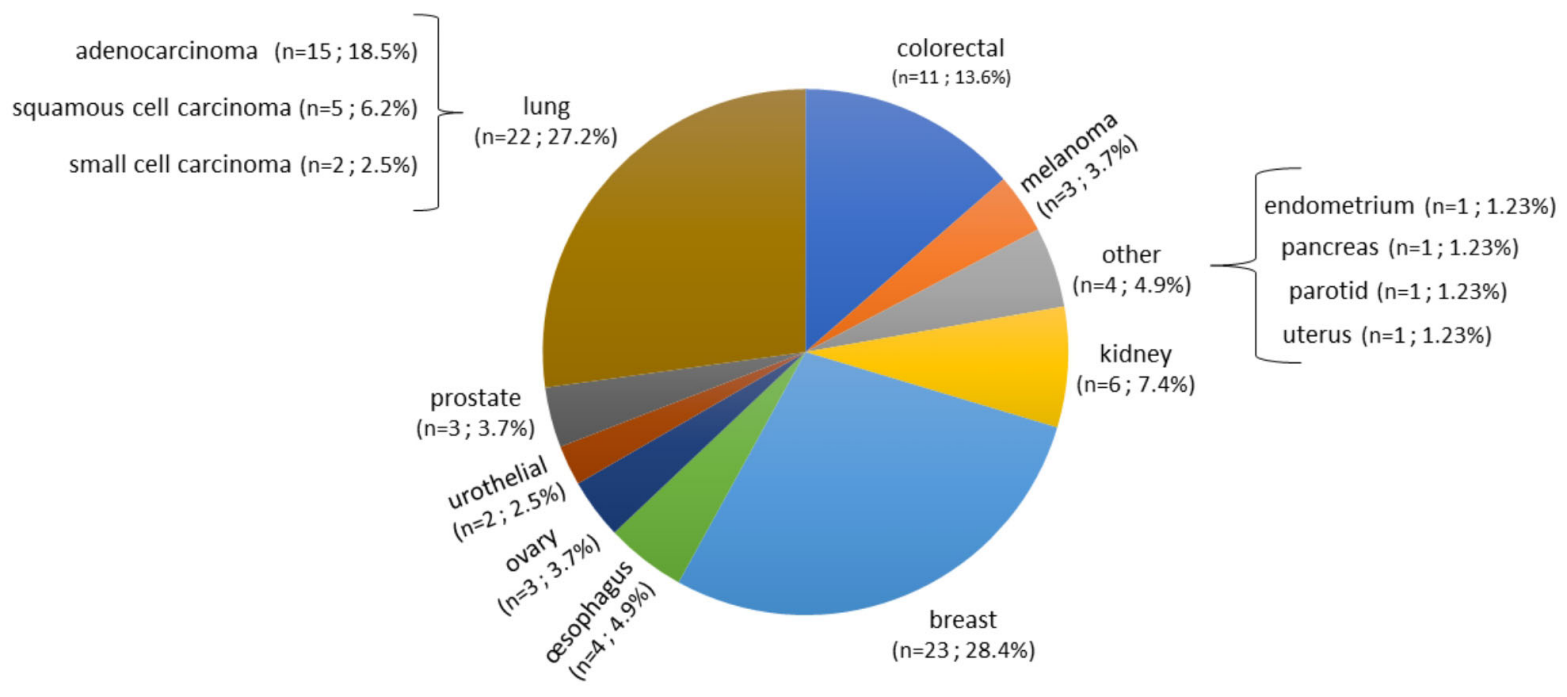
Prevalence of primary tumor types of brain metastases samples analyzed. Eighty-one samples are from patients operated on BM, from 13 different types of primary tumors as shown in the figure. Breast cancer, lung cancer, and colorectal cancer were represented at 28.4%, 27.2%, and 13.6%, respectively. The rest of the primary tumors were represented with percentage comprised between 2.5% to 7.4%.

A total of 140 samples were analyzed, including 59 samples from patients operated on glioma with the following distribution: 17 samples from WHO grade II glioma, 20 from WHO grade III glioma and 22 from WHO grade IV glioma (**Table 1**, **Figure 1**). The remaining 81 samples are from patients operated on BM, from 13 different types of primary tumors (**Table 2**). Among brain metastasis tumors histology, breast cancer and lung cancer were represented at 28.4% and 27.2%, respectively (**Figure 2**). The median percentage of cellularity of the samples analyzed was 90% (n = 140, range: 1-100%).

**Table 1:** Characteristics of glioma patients and tumor genomic alterations.

N°	Gender	Age (yrs)	Histology diagnosis	Grade	IDH 1/2	Other genetic alterations	WHO classification
1	M	25.9	astrocytoma	3	IDHm	NA	2021
2	M	59.1	glioblastoma	4	*wt*	NA	2021
3	M	60.0	astrocytoma	2	IDHm	NA	2021
4	M	26.3	oligodendroglioma	2	IDHm	1p19q co-deleted	2021
5	F	48.0	astrocytoma	2	IDHm	NA	2021
6	F	37.2	astrocytoma	2	IDHm	NA	2021
7	M	62.3	oligodendroglioma	2	IDHm	1p19q co-deleted	2021
8	F	56.9	astrocytoma	2	IDHm	1p19q not-co-deleted	2021
9	M	66.9	oligodendroglioma	2	IDHm	1p19q co-deleted	2021
10	F	56.3	astrocytoma	2	IDHm	NA	2021
11	M	24.8	oligodendroglioma	2	IDHm	1p19q co-deleted	2021
12	M	41.6	oligodendroglioma	2	IDHm	1p19q co-deleted	2021
13	F	77.2	astrocytoma	2	IDHm	1p19q not-co-deleted	2021
14	M	50.3	oligodendroglioma	2	IDHm	1p19q co-deleted	2021
15	F	35.5	astrocytoma	2	IDHm	1p19q not-co-deleted	2021
16	M	57.2	astrocytoma	2	*wt*	NA	2021
17	M	44.9	astrocytoma	2	IDHm	NA	2021
18	F	39.0	oligodendroglioma	2	IDHm	1p19q co-deleted	2021
19	F	26.2	astrocytoma	2	IDHm	NA	2021
20	F	50.4	astrocytoma	3	IDHm	NA	2021
21	M	64.5	oligodendroglioma	3	IDHm	1p19q co-deleted	2021
22	M	32.1	astrocytoma	3	IDHm	NA	2021
23	M	36.7	astrocytoma	3	IDHm	NA	2021
24	M	43.6	astrocytoma	3	*wt*	1p19q not-co-deleted	2021
25	M	51.0	astrocytoma	3	*wt*	NA	2021
26	M	61.5	astrocytoma	3	*wt*	NA	2021
27	M	37.7	astrocytoma	3	IDHm	NA	2021
28	F	36.0	astrocytoma	3	IDHm	NA	2021
29	M	31.9	astrocytoma	3	IDHm	NA	2021
30	M	34.8	astrocytoma	3	IDHm	NA	2021
31	M	37.4	astrocytoma	3	IDHm	NA	2021
32	F	46.4	oligodendroglioma	3	IDHm	1p19q co-deleted	2021
33	F	42.7	oligodendroglioma	3	IDHm	1p19q co-deleted	2021
34	F	52.6	oligodendroglioma	3	IDHm	1p19q co-deleted	2021
35	F	63.7	astrocytoma	3	IDHm	NA	2021
36	M	42.0	oligodendroglioma	3	IDHm	1p19q co-deleted	2021
37	M	63.2	oligodendroglioma	3	IDHm	1p19q co-deleted	2021
38	F	36.8	astrocytoma	3	IDHm	NA	2021
39	F	43.7	astrocytoma	3	IDHm	NA	2021
40	F	58.1	astrocytoma	4	IDHm	NA	2021
41	M	29.8	astrocytoma	4	IDHm	NA	2021
42	M	40.9	astrocytoma	4	IDHm	NA	2021
43	M	37.5	glioblastoma	4	*wt*	NA	2021
44	M	55.1	glioblastoma	4	*wt*	NA	2021
45	M	48.8	glioblastoma	4	*wt*	NA	2021
46	M	62.0	glioblastoma	4	*wt*	NA	2021
47	M	71.7	glioblastoma	4	*wt*	NA	2021
48	F	67.1	glioblastoma	4	*wt*	NA	2021
49	M	73.3	glioblastoma	4	*wt*	NA	2021
50	M	51.1	glioblastoma	4	*wt*	NA	2021
51	M	66.0	glioblastoma	4	*wt*	NA	2021
52	M	43.4	glioblastoma	4	*wt*	NA	2021
53	F	56.1	glioblastoma	4	*wt*	NA	2021
54	M	85.2	glioblastoma	4	*wt*	NA	2021
55	F	73.3	glioblastoma	4	*wt*	NA	2021
56	F	68.8	glioblastoma	4	*wt*	NA	2021
57	F	51.8	glioblastoma	4	*wt*	NA	2021
58	M	72.5	glioblastoma	4	*wt*	NA	2021
59	M	68.8	glioblastoma	4	*wt*	NA	2021

NA, not available; wt, wildtype; M, Male; F, Female.

**Table 2:** Brain Metastasis histology diagnosis.

N°	Gender	Age (yrs)	Histology diagnosis	Primary tumor
1	M	59.8	BM from pancreatic adenocarcinoma	pancreas
2	M	74.5	BM from poorly differentiated carcinoma of esophagus	esophagus
3	M	60.6	BM from oesophageal adenocarcinoma	esophagus
4	M	67.8	BM from oesophageal adenocarcinoma	esophagus
5	F	70.3	BM from urothelial carcinoma	urothelial
6	F	56.5	BM from urothelial carcinoma	urothelial
7	M	61.2	BM from gastric adenocarcinoma	esophagus
8	F	62.4	BM from endometrial adenocarcinoma	endometrium
9	M	72.5	BM from rectal Lieberkhunian adenocarcinoma	Rectum
10	M	73.6	BM from digestive adenocarcinoma	rectum
11	F	66.2	BM from ovarian adenocarcinoma	ovary
12	M	78.2	BM from ovarian serous carcinoma	ovary
13	M	69.2	BM from prostatic adenocarcinoma	prostate
14	M	78.2	BM from prostatic adenocarcinoma	prostate
15	M	73.0	BM from prostatic adenocarcinoma	prostate
16	F	42.4	BM from malignant melanoma	melanoma
17	M	45.5	BM from malignant melanoma	melanoma
18	F	43.9	BM from malignant melanoma	melanoma
19	M	73.0	BM from colonic adenocarcinoma	colon
20	F	57.8	BM from colonic adenocarcinoma	colon
21	F	67.3	BM from colonic adenocarcinoma	colon
22	F	74.8	BM from colonic adenocarcinoma	colon
23	M	77.5	BM from colonic adenocarcinoma	colon
24	F	52.5	BM from colonic adenocarcinoma	colon
25	M	63.6	BM from colonic adenocarcinoma	colon
26	M	59.3	BM from colonic adenocarcinoma	colon
27	M	55.4	BM from colonic adenocarcinoma	colon
28	M	67.5	BM from conventional renal cell adenocarcinoma	kidney
29	M	73.9	BM from renal cell carcinoma	kidney
30	M	78.0	BM from clear cell renal carcinoma	kidney
31	F	76.5	BM from conventional renal cell adenocarcinoma	kidney
32	M	47.9	BM from conventional renal cell adenocarcinoma	kidney
33	M	56.9	BM from conventional renal cell carcinoma with sarcomatoid features	kidney
34	M	71.6	BM from parotid adenocarcinoma	paritid
35	F	33.0	BM from breast adenocarcinoma	breast
36	F	51.6	BM from breast adenocarcinoma	breast
37	F	49.7	BM from breast adenocarcinoma	breast
38	F	78.1	BM from breast adenocarcinoma	breast
39	F	64.0	BM from breast adenocarcinoma	breast
40	F	52.6	BM from breast adenocarcinoma	breast
41	F	76.4	BM from breast adenocarcinoma	breast
42	F	40.7	BM from breast adenocarcinoma	breast
43	F	73.5	BM from breast adenocarcinoma	breast
44	F	58.6	BM from breast adenocarcinoma	breast
45	F	73.5	BM from breast adenocarcinoma	breast
46	F	58.6	BM from breast adenocarcinoma	breast
47	F	66.6	BM from breast adenocarcinoma	breast
48	F	57.0	BM from breast adenocarcinoma	breast
49	F	70.5	BM from breast adenocarcinoma	breast
50	F	57.6	BM from breast adenocarcinoma	breast
51	F	36.9	BM from breast adenocarcinoma	breast
52	F	73.1	BM from breast adenocarcinoma	breast
53	F	65.3	BM from breast adenocarcinoma	breast
54	F	53.9	BM from breast adenocarcinoma	breast
55	F	51.0	BM from breast adenocarcinoma	breast
56	F	77.3	BM from poorly differentiated squamous cell lung carcinoma	lung
57	M	85.7	BM from small cell carcinoma-like neuroendocrine lung carcinoma	lung
58	M	65.7	BM from moderately differentiated squamous cell lung carcinoma	lung
59	M	85.7	BM from moderately differentiated non-keratinizing squamous cell lung carcinoma	lung
60	F	74.8	BM from well-differentiated keratinizing squamous cell lung carcinoma	lung
61	F	66.4	BM from lung adenocarcinoma	lung
62	M	72.9	BM from lung adenocarcinoma	lung
63	F	64.6	BM from papillary-type lung adenocarcinoma	lung
64	M	67.1	BM from lung adenocarcinoma	lung
65	M	57.0	BM from lung adenocarcinoma	lung
66	F	66.1	BM from lung adenocarcinoma	lung
67	F	66.0	BM from lung adenocarcinoma	lung
68	F	70.2	BM from lung adenocarcinoma	lung
69	F	63.4	BM from lung adenocarcinoma	lung
70	M	76.0	BM from lung adenocarcinoma	lung
71	F	50.3	BM from lung adenocarcinoma	lung
72	F	67.1	BM from neuroendocrine carcinoma of small cell lung carcinoma	lung
73	M	79.0	BM from lung adenocarcinoma	lung
74	F	77.4	BM from lung adenocarcinoma	lung
75	F	62.3	BM from lung adenocarcinoma	lung
76	M	65.4	BM from anaplastic small cell lung carcinoma	lung
77	M	49.2	BM from lung adenocarcinoma	lung
78	F	73.4	BM from ovarian adenocarcinoma	ovary
79	F	59.9	BM from breast adenocarcinoma	breast
80	F	76.7	BM from breast adenocarcinoma	breast
81	F	55.8	BM from uterine adenocarcinoma	uterus

The patients’ characteristics are summarized in **Table 3**. In the overall population analyzed, we had as many men as women. The average age at the time of surgery is 58.6 ± 14.5 with a median age of 60.9 [rank: 24.8 – 85.7]. The demographic characteristics of the two cohorts: gliomas and BM are also detailed in **Table 3**.

**Table 3:** Demographic characteristics of the two cohorts: glioma and BM.

	Total population n = 140	Gliomas n = 59	Brain metastasis n = 81
Characteristics	Value	Value	Value
**Age at surgery (yrs)** **Median** **Mean +/- SD** **Range**	**60.9** **58.6 +/- 14.5** **24.8 – 85.7**	**50.4** **50.6 +/- 14.7** **24.8 – 85.2**	**66.0** **64.5 +/- 11.2** **33.0 – 85.7**
**Gender** **Males** **Females**	**70 (50.0%)** **70 (50.0%)**	**37 (62.7%)** **22 (37.3%)**	**33 (40.7%)** **48 (59.3%)**

### 3.2 Baseline characteristics of the panel

Several groups have published on multiplex amplicon approaches that specifically target fusions across known break points (29–31). The main advantages of such amplicon approaches include lower-input requirements, potentially increased sensitivity attributable to extensive amplification, shorter technical time for the assay, and reduced complexity for data analysis.

In accordance with our pre-defined quality control parameter, the sequencing data was processed carefully to make sure the correctness of the analysis. Quality control was an integral part of our strategy where specific metrics such as mean RNA read length (> 60 bp) and mean raw accuracy (> 99%) are used as indicators of sequencing. Besides, the minimum threshold of total sequencing reads (> x 21,000,000) was set to ensure the adequacy of coverage for the reliable analysis. The quality of sequencing data was assessed by analyzing the number of mapped reads by analyzing the number of mapped reads for each sample. Most samples exhibited consistent and high levels of mapped reads, ranging from approximately 150,000 to 400,000 reads. This consistency indicated successful library preparation, sequencing, and alignment processes for most the samples.

**Figure 3:**
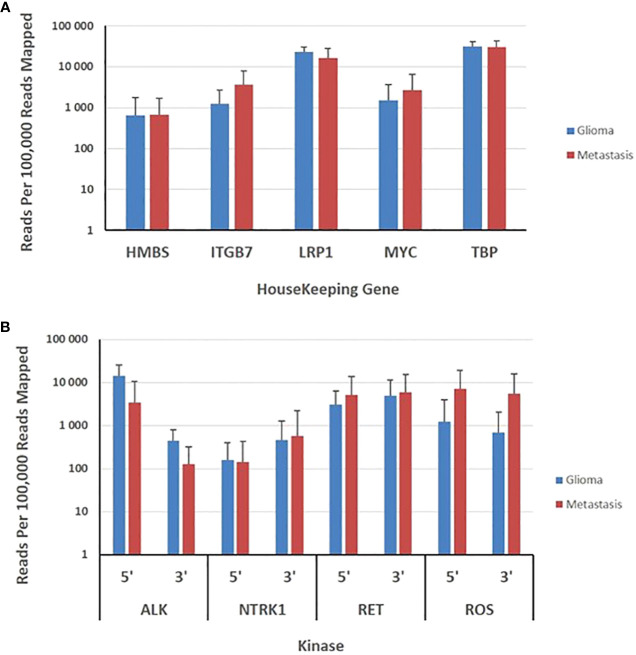
Evaluation of brain gliomas and brain metastasis tissues specimens. **(A)**, Expression levels of five housekeeping genes are depicted as the normalized number of reads per 100,000 reads mapped to assay amplicons for each transcript and represent the means ± SD derived from 59 gliomas and 81 brain metastasis tissue specimens. **(B)**, Expression levels of 5’ and 3’ exons in 4 kinases are depicted as the means ± SD of the normalized sequence reads observed in 59 gliomas and 81 brain metastasis tissue specimens.

Transcripts derived from five housekeeping genes ranged from a mean of approximately 700 (*HBM5*) to 15,000 (*TBP*) reads per 100,000 mapped reads. The expression of housekeeping genes was evaluated in 59 gliomas and 81 metastases. The panel revealed no significant variability in expression of housekeeping genes between metastatic and primary tumors (**Figure 3**). The 3’/5’ read ratio was measured for 4 kinases (ALK, NTRK, RET, ROS) on the panel. The observed reads in the gliomas and metastasis samples ranged from approximately 100 to 10,000 mapped reads per 100,000 mapped reads (**Figure 3**). The 3’/5’ ratios were close to one for the four kinases (**Figure 3**).

To determine the limit of detection of the RNA Oncomine assay to detect the fusion transcripts, SeraSeq Fusion RNA Mix v4 (Seracare Life Sciences Inc.) was used, which contains 17 clinically relevant *NTRK* fusions, including high prevalence fusions, such as *TPM3*-*NTRK1* and *FGFR3-TACC3, ETV6*-*NTRK3*, as well as less common fusions, such as *TFG*-*NTRK1*, *CCDC6-RET*, *CD74-ROS1*, *FGFR3-BAIAP2L1*, *KIF5B*-*RET*, *EML4*-*ALK*, *LMNA-NTRK1, NCOA4-RET*, *PAX8*-*PPARG1*, *SLC34A2*-*ROS1*, *SLC45A3*-*BRAF*, *TMPRSS2-ERG*, *EGFR variant III* and *MET ex 14* skipping. All 17 RNA fusions were detected by the panel RNA Oncomine™ FOCUS Assay (OFA) with 560, 1454, 455, 1014, 194, 222, 446, 391, 142, 37, 100, 118, 299, 460, 85, 126, and 264 mapped reads, respectively.

Based on these results, we defined a high-confidence gene fusion meeting the following criteria: a minimum threshold of 20 fusion reads per 100,000 reads mapped to assay amplicons (0.01%) and clear evidence that the reads spanned the target fusion. We further required a library yield at least 100,000 mapped reads for a specimen to be called truly negative.

### 3.3 Fusion gene detection in tumor specimens

**Figure 4:**
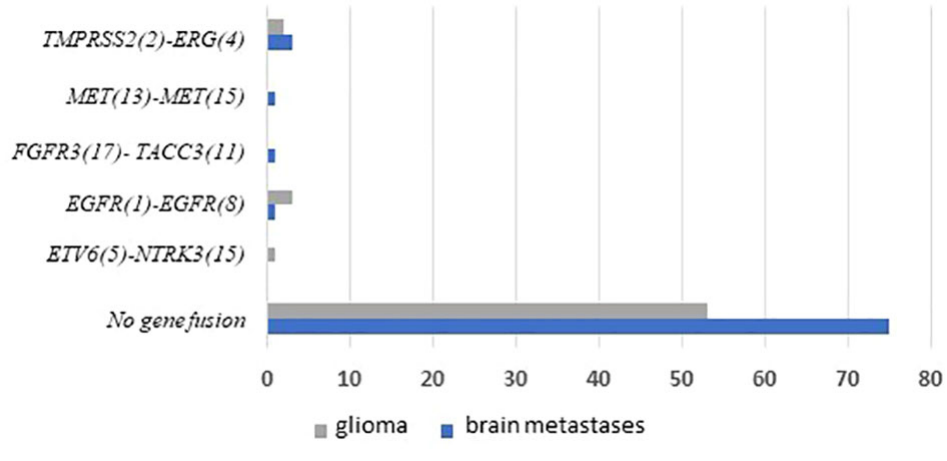
Frequencies and distributions of fusions for all brain gliomas and brain metastases with 140 cases analyzed. The RNA sequencing analysis of the two cohorts ‘gliomas and BM demonstrated samples with gene rearrangements that involve *NTRK3*, *FGFR3*, *ERG*, *EGFR*, or *MET* genes in seven samples (5%). Different types of alterations are found such as *ETV6 (* 5)- *NTRK3 (* 15) (1/59 gliomas – 1.69%), *TMPRSS (* 2)*-ERG* (4) fusion (5/140 – 3.6%), *FGFR3 (* 17)-*TACC3* (11) fusion(1/140 – 0.7%), *EGFR (* 1)*-EGFR* (8) RNA transcripts (4/59 gliomas - 6.78%); *MET (* 13)*-MET* (15) RNA transcripts (1/140 – 0.7%).

**Figure 5:**
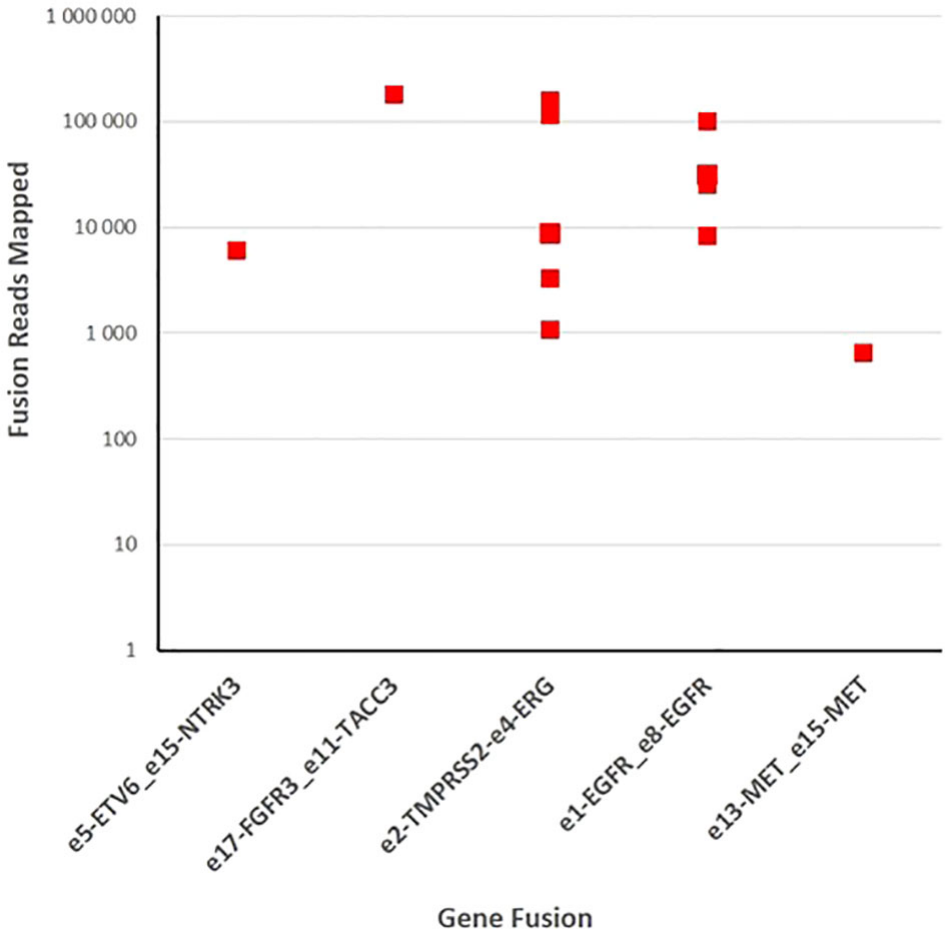
Fusion gene detection in tumor specimens. One hundred forty samples of gliomas and brain metastases were sequenced with fusion gene amplicon panel. Twelve samples were found to harbor gene rearrangements that involve *NTRK3*, *FGFR3*, *ERG, EGFR, and MET*.

**Figure 6:**
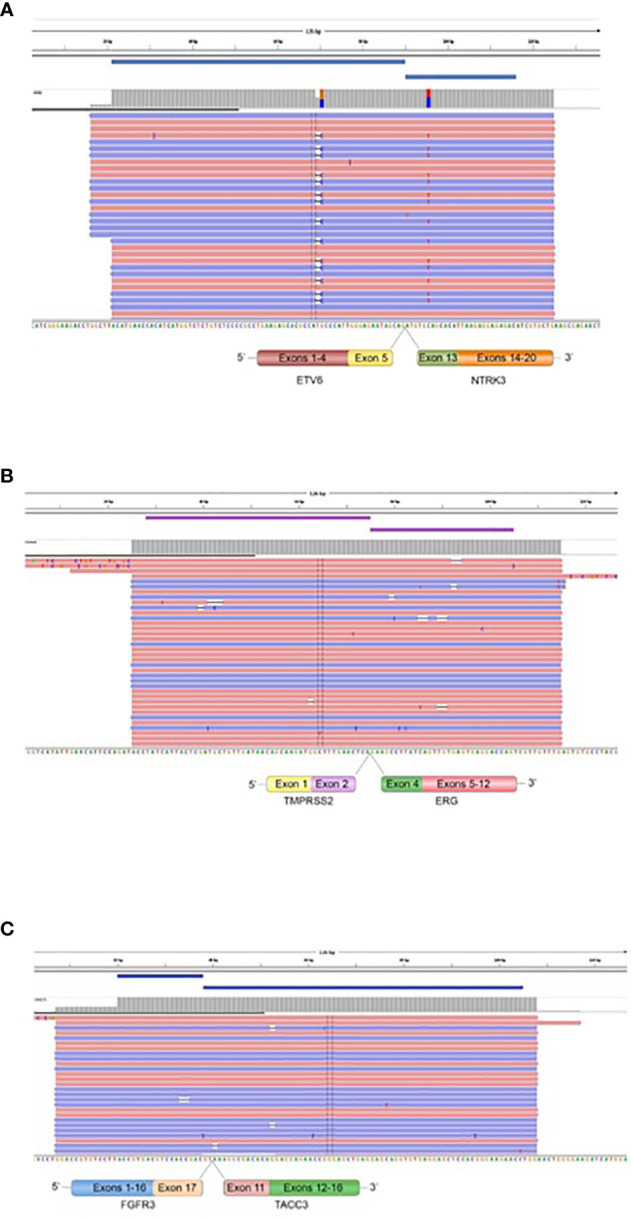
Fusions confirmed by targeted Next Generation Sequencing. The presence of *ETV6*::*NTRK3* in oligodendroglioma (grade II), IDH-mutated and 1p19q co-deleted **(A)**, *TMPRSS2*::*ERG* in prostatic carcinoma BM, endometrium BM, and oligodendroglioma (grade II), IDH-mutated and 1p19q co-deleted **(B),** and *FGFR3*::*TACC3* in breast BM **(C)** is demonstrated by fusion amplicon panel. Visualization of RNA sequencing reads supports the fusions junctions between *ETV6* exon 5 and *NTRK3* exon 15, *TMPRSS2* exon 2 and *ERG* exon 4, and *FGFR3* exon 17 and *TACC3* exon 11.

**Figure 7:**
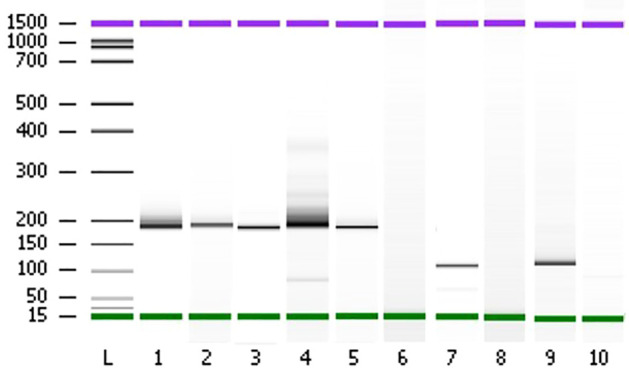
Polyacrylamide gel separation of the *TMPRSS2 (* 2)::*ERG* (4), *FGFR3 *(17)::*TACC3* (11)*, ETV6 (* 15)::*NTRK3* (15) fusion specific RT-PCR amplicons. Samples (1–5) show one major band corresponding to the variant of the fusion between *TMPRSS2* exon 2 and *ERG* exon 4. Sample 7 shows a variant connecting exon 5 of *ETV6* and exon 15 of *NTRK3*. Sample 9 shows one variant fusing together *FGFR3* exon 17 and *TACC3* exon 11. The sample of total RNA from patient 80 which didn’t show any fusion events was used as negative controls (lanes 6, 8, and 10). L; Ladder on base pair (bp).

We performed targeted NGS on a subset (n = 140) of tumors. This subset presents an average of tumor content > 80%. The RNA sequencing analysis of 140 samples of the two cohorts ‘gliomas and BM revealed samples with gene rearrangements that involve *NTRK3*, *FGFR3*, *ERG*, *EGFR*, or *MET* genes in seven samples (5%) (**Figure 4**). In one tumor of 59 glioma specimens, one rearrangement with 6225 fusion reads mapped fusing exon 5 of the gene *ETV6* to exon 15 of *NTRK3* was identified (**Figure 5**, **Table 4**) in oligodendroglioma–grade II, IDH-mutated and 1p19q co-deleted at frequency of 1.69%. The *TMPRSS* (2)*::ERG* (4) fusion (5/140 – 3.6%) was the most frequently gene fusions detected with 1101, 3306, 8918, 116645 and 162560 fusion mapped reads (**Figure 5**), two of them were identified in two patients operated on WHO grade II gliomas, both of whom had a history of other cancers (adenocarcinoma of the lower esophagus and papillary adenocarcinoma of thyroid), two were identified in two patients operated on for prostate carcinomas brain metastasis and one was identified in patient operated on for endometrium carcinomas brain metastasis. A rearrangement fusing exon 17 of the gene *FGFR3* to exon 11 of *TACC3* gene (1/140 – 0.7%) (**Table 2**) was detected with 184470 fusion mapped reads in patient operated for breast carcinoma brain metastasis (**Figure 5**). The high numbers of NGS mapped reads suggest the higher expression of the fusion transcripts in the tumor tissues (**Table 4**). The characteristics of the corresponding samples identified with fusions are summarized in **Table 4**. The visualization of RNA sequencing reads supports the fusion between the *ETV6* exon 5 and *NTRK3* exon 15 (**Figure 6**), the *TMPRSS* exon 2 and *ERG* exon 4 (**Figure 6**), and *FGFR3* exon 17 and *TACC3* exon 11 (**Figure 6**). We used specific PCR primers flanking the breaking points of *ETV6* (exon 5) and *NTRK3* (exon 15), *FGFR3* (exon 17) and *TACC3* (exon 11), and *TMPRSS2* (exon 2) and *ERG* (exon 4) to realize RT-PCR analysis in order to confirm and validate the identified fusions events by targeted RNA sequencing (**Figure 7**).

**Table 4:** Identified fusions and characteristics of the corresponding samples.

Identified fusions	NGS	N° of fusions	Gender	Age (yrs)	Histology
	sequencing reads	(n; %)			
***NTRK* gene fusion**					
**NTRK1**		**0 (0%)**			
**NTRK2**		**0 (0%)**			
**NTRK3**		**1 (1.69%)**			
*ETV6* (5)*::NTRK3* (15)	6225		M	66.8	Oligodendroglioma (grade II) IDH-mutant & 1p19q co-deleted
**FGFR3**		**1 (0.7%)**			
					
*FGFR3* (17)*::TACC3* (11)	184470		F	76.4	Breast BM
**TMPRSS2**		**5 (3.6%)**			
*TMPRSS2 (* 2 *)::ERG (* 4 *)*	116645		M	73	Prostatic BM
*TMPRSS2 (* 2 *)::ERG (* 4 *)*	1101		M	69.2	Prostatic BM
*TMPRSS2 (* 2 *)::ERG (* 4 *)*	3306		F	62.4	Endometrium BM
*TMPRSS2 (* 2 *)::ERG (* 4 *)*	162560		M	26.3	Oligodendroglioma (grade II)* IDH-mutant & 1p19q co-deleted
*TMPRSS2 (* 2 *)::ERG (* 4 *)*	8918		M	62.3	Oligodendroglioma (grade II) IDH-mutant & 1p19q co-deleted **

*history of thyroid papillary adenocarcinoma; **history of adenocarcinoma of the lower esophagus; M, male; F, female.

### 3.4 Exons skipping transcripts

**Figure 8:**
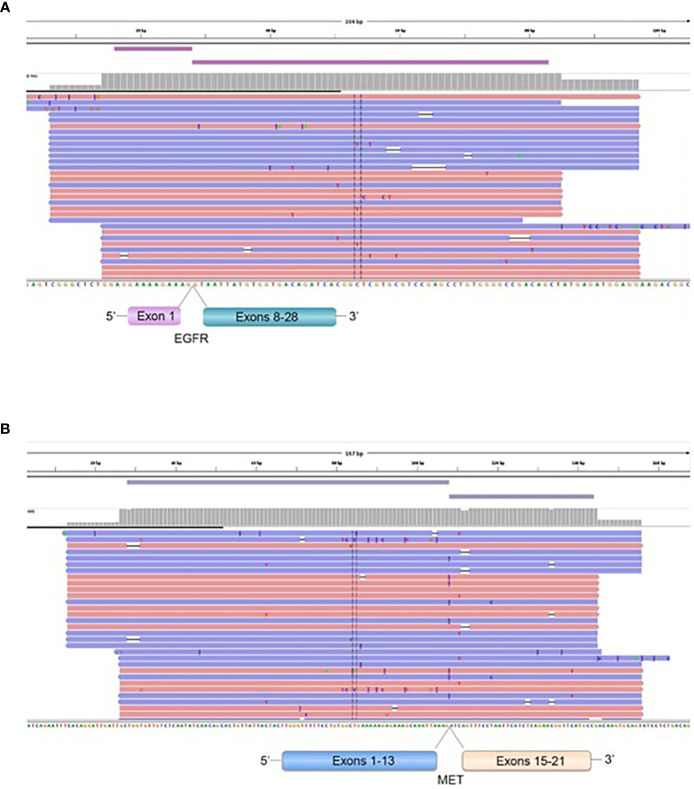
Representation of the *EGFR* (1)-*EGFR* (8) in a GBM patient **(A)**, and *MET* (13)-*MET* (15) **(B)**, fusions transcripts. Visualization of RNA sequencing reads by Next Generation Sequencing demonstrates the fusion junction between *EGFR* exon 1*-EGFR* exon 8 and *MET* exon 13-*MET* exon 15.

Four patients operated for GBM *IDH* wild-type (grade IV) showed *EGFR* exons 2-7 skipping mRNA with variable levels of the number of fusion reads at 8453, 25600, 31705, and 10214 reads, respectively, to generate *EGFR* (1)*-EGFR* (8) RNA transcripts (4/59 gliomas - 6.78%) (**Figure 5**, **Table 5**). One patient operated for pancreas carcinoma BM was identified harboring *METex14* skipping with 667 reads to generate *MET* (13)*-MET* (15) RNA transcripts (1/140 – 0.7%) (**Figure 5**, **Table 5**). The RNA sequence analysis supports the fusion of exon 1 to exon 8 for the *EGFR* gene (**Figure 8**) and the fusion of exon 13 to exon 15 for the *MET* gene (**Figure 8**). In contrast to DNAseq in which detection limits rely strictly on the prevalence of cancer cells present in the tumor sample, additional variation must be considered when dealing with RNAseq data, including expression of fusion genes, which can be highly variable. As can be observed in **Table 5** and **Figure 5**, the number of reads of each alteration is highly variable between tumor samples meaning high variability of expression of these transcripts which, could be a consequence of *in vivo* conditions and microenvironment of each tumor sample. The more the number of reads is high the more the RNA transcript fusion is present.

**Table 5:** Exons skipping transcripts and characterization of the corresponding samples.

Identified transcripts	NGS	N° of exons skipping transcripts	Gender	Age (yrs)	Histology
	Sequencing reads	(n; %)			
***EGFR***		**4 (6.7%)**			
*EGFR (* 1 *) – EGFR (* 8 *)*	8453		F	56.1	GBM, IDH-wt
*EGFR (* 1 *) – EGFR (* 8 *)*	102141		F	67.1	GBM, IDH-wt
*EGFR (* 1 *) – EGFR (* 8 *)*	31705		F	66.7	GBM, IDH-wt
*EGFR (* 1 *) – EGFR (* 8 *)*	25600		M	68.7	GBM, IDH-wt
***MET***		**1 (0.7%)**			
*MET* (13) *– MET* (15)	667		M	59.8	Pancreas BM

In glioma cohort: 89.8% no gene fusion; 1.7% ETV6 (5)::NTRK3 (15) fusion; 5.1% of EGFR exons 2-7 skipping to generate EGFR (1)-EGFR (8) transcripts; 0% FGR3 (17)::TACC3 (11) fusion; 0% of MET exon 14 skipping transcripts; 3.4% TMPRSS2 (2)::ERG (4) fusion; M, male; F, female; GBM, Glioblastoma; IDH-wt, IDH-wildtype.

In BM cohort: 92.6% no gene fusion; 0% of ETV6 (5)::NTRK3 (15) fusion; 1.2% of EGFR (1)::EGFR (8) fusion; 1.2% of FGR3 (17)::TACC3 (11) fusion; 1.3% of Met ex14 skipping to generate MET (13)-MET (15) transcripts; 3.8% of TMPRSS2 (2)::ERG (4) fusion.

### 3.5 Brain tumors and brain metastasis molecular testing in clinics

**Figure 9:**
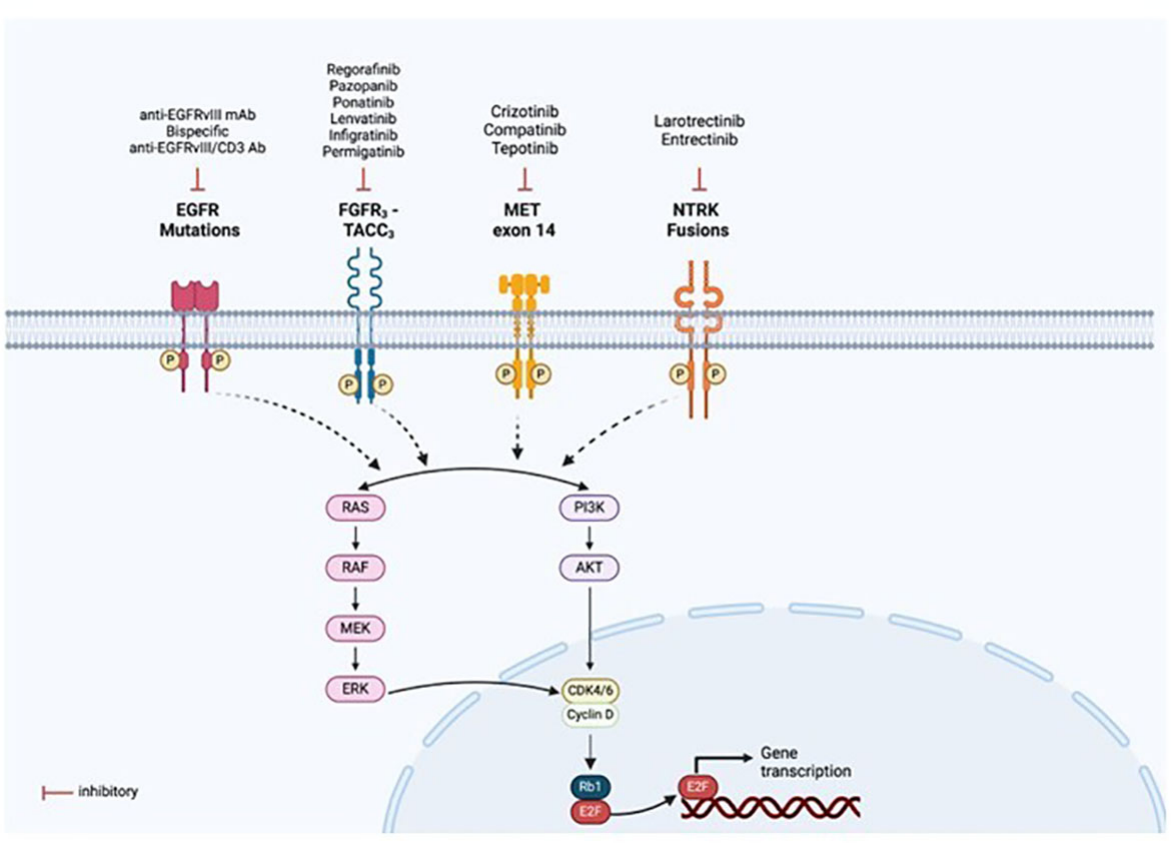
Targeted therapies available for the fusion proteins detected. Mutations or rearrangements in receptor tyrosine kinases (RTKs) result in constitutive activation of the kinase domain even in the absence of ligand binding and aberrant downstream signalling, leading to activation of multiple pathways, notably PI3K/AKT and RAS/MAPK. This allows for drug designs and development of targeted therapies specific to each oncogenic alteration, as illustrated in the figure. Created with BioRender.com.

Identifying molecular oncogenic drivers is crucial for precision oncology. Chromosomal rearrangements could result in gene fusions that lead to the expression of oncoproteins. When the fusion involves a receptor tyrosine kinase (RTK), the tyrosine kinase domain (TKD) is activated—often constitutively and ligand-independent—and downstream effectors of the receptor receive constant signaling, causing uncontrolled cell growth and invasiveness (32). Then, the tumor cell becomes dependent on this oncogenic RTK to maintain its malignant properties. This dependency, also called “oncogene addiction,” can be therapeutically approached with drugs that inhibit the activity of the oncoprotein (**Figure 9**). Currently, most RTKs inhibitors are designed to prevent either the binding of the ligand—often by using monoclonal or bispecific antibodies, and antibody-drug conjugates—or the binding of the ATP to the catalytic domain—mostly with small molecules (**Figure 9**). According to clinical guidelines, an upfront genomic profiling test should be a priority to detect targetable oncogenic alterations in gliomas and brain metastases. Different diagnostic methods, including IHC, FISH, reverse transcriptase PCR, and DNA/RNA-based NGS, can be used to detect gene fusions. However, based on the increasingly frequent need for a comprehensive genomic evaluation, NGS panels are becoming the preferred approach.

## 4 Discussion

Tumors often utilize a recurring mechanism to achieve over-expression of oncogenic kinases, which involves fusing kinases to genes that are highly expressed in tumors or the tissue of tumor-origin (28, 33–35). Since then, targeting the fusion genes has become the primary treatment option for fusion-positive patients, given that tumors with those fusions usually exhibit strong oncogenic addiction (26, 36). In our study, we observed an *ETV6* (5)*::NTRK3* (15) fusion in 1.69% of diffuse IDH-mutated 1p19q co-deleted oligodendroglioma (grade II), accounting for a significant portion of glioma by this mechanism. Although a prior study observed *NTRK1* fusions at an incidence of 1% in glioblastoma (37), we didn’t find any *NTRK1* fusion in our series of 59 tumor samples from patients operated for low and high-grade gliomas. *NTRK* fusions are present in a small percentage of gliomas/neuroepithelial tumors, with an estimated incidence ranging from 0.55% to 2% (13, 14, 38, 39). However, the incidence may be higher in certain groups, such as up to 5.3% in pediatric high-grade gliomas (HGG) (15), 4% of diffuse intrinsic pontine gliomas (DIPG), and up to 40% of non-brainstem HGG in patients younger than 3 years old (33). Gliomas with *NTRK* fusions have been previously reported to possess co-occurring genetic alterations such as *IDH* (13, 14, 31)*, H3.3 K27M* (11)*, H3F3A* (40)*, EGFR* amplification (13), *EGFRvIII* (13)*, PTEN* (13), *CDKN2A/2B* deletion (11, 37), *CDKN2C* deletion (37), *TP53* mutations/inactivation (13), and *ATRX* (41), among others (13). A variety of *NTRK* fusion types (*NTRK1*, *NTRK2*, and *NTRK3*) have also been described in pediatric high-grade gliomas (11). Several downstream signaling pathways, including SHC-RAS-MAPK, PI3K-AKT, PLCγ-PKC, or STAT3, are activated by the three wildtype TRK family members (42), suggesting that most NTRK fusions would use many of these downstream signaling cascades as full-length receptors. It has been demonstrated that the TRK oncogenes induce a transformation of NIH-3T3 fibroblasts and thyroid epithelial cells (43). Similarly, mammary epithelial cells was shown to be transformed using *ETV6* (5)*::NTRK3* (15) fusion (44). Experiments with *ETV6*::*NTRK3* fusion showed that the fusion protein signals mainly through RAS/MAPK but also activates PI3K/AKT/mTOR. Activation of both pathways might result in a potency oncogene to stimulate proliferation and inhibits apoptosis (45). Taken together, these data support that the *ETV6*::*NTRK3* fusion present in the oligodendroglioma (grade II) could be a driver to promote tumor growth *in vivo*, suggesting that *NTRK* inhibitors may be a valuable therapeutic option to delay or avoid the need for radiotherapy in this population.

The *NTRK* fusion has been previously targeted by different drugs among which the most used are the first-in class TRK-targeting inhibitors are Larotrectinib (selective TRK inhibitor) and entrectinib (pan-TRK, ROS1 and ALK inhibitor). In 2018, the United States Food and Drug Administration (FDA) approved larotrectinib, a highly specific inhibitor of all three TRK proteins, for adult and pediatric patients with solid tumors (46). Larotrectinib’s approval was based on results from three multi-center clinical studies (a phase 1 trial (NCT02122913), SCOUT (NCT02637687), and NAVIGATE (NCT02576431)) (10, 47). According to a clinical trial involving solid tumors positive for NTRK fusion, Larotrectinib showed an overall response rate of 79.1%, with a median duration of response lasting 35.2 months, and a progression-free survival of 28.2 months (48). In 2019, entrectinib, a multi-kinase inhibitor targeting TRK proteins, c-ROS oncogene 1 (ROS1), and anaplastic lymphoma kinase (ALK) was approved by the FDA for adult and pediatric patients (>12 years) with the same indication as larotrectinib (49). Entrectinib’s approval was based on results of the multi-center trials; ALKA-372-001, STARTRK-1, and STARTRK-2 (50, 51). These trials revealed a response rate of 57% in patients with TRK fusion-positive solid tumors across 10 different tumor types (50, 51). The use of inhibitors targeting TRK is associated with high response rates regardless of tumor histology and patient age (1, 2, 52).

Several studies demonstrate the activity of Larotrectinib in TRK fusion-positive primary CNS tumors regardless of histology, which confirms its capacity for blood-brain barrier penetrance as reported previously by the response observed in metastases of extra-cranial tumors to CNS (48, 53). Larotrectinib demonstrated rapid and durable responses in TRK fusion-positive primary CNS tumors, and responses were seen in patients with low- and high-grade gliomas as well as non-gliomas (54). The intracranial efficacy of Larotrectinib has been demonstrated in TRK fusion-positive tumors that have metastasized to the brain (48, 53). These results further support expanded testing for actionable therapeutic targets, including NTRK gene fusions, in patients with primary adult and pediatric CNS tumors and BM.

Members of the receptor tyrosine kinase gene family including *EGFR*, *MET*, *PDGFRA*, and *FGFR3* have been known to be heavily involved in the initiation and progression of glioblastoma (20, 55, 56). In the present study, we also identified the presence of *FGFR3 *(17)::*TACC3* (11) fusion in breast carcinomas BM. The constitutively active signal of FGFR3 also transduces via the RAS/MAPK pathway (57). Previously, Singh et al. reported three fusions of *FGFR*::*TACC* in 97 glioblastoma examined (20). They demonstrated that the fusion protein has oncogenic activity when introduced into astrocytes and treatment with FGFR inhibitor extends the survival of mice harboring intracranial *FGFR*::*TACC*-initiated glioma. Previously, Parker et al. reported that 4 out of 48 glioblastoma samples harbored the *FGFR3*::*TACC3* fusion and remarkably showed that the tumorigenic *FGFR3*::*TACC3* gene fusion escapes miR-99a regulation in glioblastoma due to the loss of the 3’-UTR of *FGFR3* (57). FGFR3 is very lowly expressed in normal brain but is highly expressed in fusion positive glioblastoma that is likely due to the loss of microRNA regulation (58, 59). The 3’-UTR of the *FGFR3* gene is negatively controlled by microRNAs in the normal brain. In the fusion gene, the region is lost and *FGFR3* can no longer be controlled by *mir-99a* (58). The *FGFR3*::*TACC3* fusion was detected in one out of 72 samples of glioblastoma in the Ivy Center cohort (60), and 2 of 161 samples in the TCGA cohort. *FGFR* genes fusions are also identified in other cancers, including bladder cancer, cholangiocarcinoma, squamous lung cancer, thyroid cancer, oral cancer, head and neck squamous cell carcinoma, and prostate cancer (25, 61). Clinical trials with FGFR inhibitors in brain tumors are being conducted (23, 24). The FGFR inhibitor ponatinib demonstrate an improved therapeutic activity of temozolomide on DIPG cells derived from patient in *in vitro* study (62).

The *EGFR* gene is frequently amplified and rearranged in malignant gliomas with an expression of oncogenic deletion mutants (56). In this study, we found four glioblastoma harboring *EGFR* exons 2-7 skipping, also known as *EGFRvIII*, which is constitutively auto phosphorylated and inefficiently down regulated (63).

In this study, we also identified a METex14 skipping within the RNA in pancreas carcinomas BM resulting in an in-frame deletion of the juxta-membrane domain, which normally is a negative regulator of the kinase catalytic activities. Aberrant MET/HGF regulation is observed in a wide variety of human cancers with a dysregulated proliferative and invasive signaling program, epithelial-to-mesenchymal transition, cell motility/migration, angiogenesis, invasion, and metastasis. The *MET*ex14 mutations were identified in 221 positive cases (0.6%) out of 38,028 profiled tumors in the largest tumor genomic profiling cohort performed for MET alteration (64).

This study showed also the presence of *TMPRSS2* (2)::*ERG* (4) fusions in prostate carcinomas BM, endometrium carcinomas BM, and oligodendroglioma (grade II), IDH-mutated and 1p19q co-deleted. Studies have shown that the androgen signaling pathway plays a role in facilitating the formation of the *TMPRSS2*::*ERG* gene fusion, which is present in approximately 50% of prostate carcinomas. This pathway induces proximity of the *TMPRSS2*::*ERG* genomic loci, which are then exposed to gamma irradiation, resulting in DNA double-strand breaks (65). Ongoing clinical trials are further evaluating the prognostic and predictive value of *ERG* fusions in prostate cancer patients at different stages of the disease or during treatment (e.g., trials evaluating the AR signaling inhibitors enzalutamide and apalutamide, PSMA theranostics, brachytherapy; see ClinicalTrials.gov for reference) and include the analysis of ERG fusion status both in primary and secondary outcome measures.

## 5 Conclusion

Genomic rearrangements are the primary way fusions arise in gliomas. Although clinically relevant fusions are rare, RNA-Seq of low- and high-grade glioma samples is a crucial molecular biology technique to discover patient-specific fusions that could guide personalized treatment. *FGFR1*-3 fusions, like *NTRK1-3*, offer a therapeutic option for current and forthcoming FGFR inhibitors across various patient subgroups.

## Data availability statement

The raw data supporting the conclusions of this article will be made available by the authors, without undue reservation.

## Ethics statement

The studies involving humans were approved by COS (Comité d’Orientation Scientifique) de la Direction Recherche et Enseignement Ramsay Santé. The studies were conducted in accordance with the local legislation and institutional requirements. The participants provided their written informed consent to participate in this study.

## Author contributions

PM: Conceptualization, Funding acquisition, Validation, Writing – original draft, Writing – review & editing. CC: Writing – review & editing. EB: Formal analysis, Resources, Validation, Writing – original draft, Writing – review & editing. NB: Methodology, Validation, Writing – review & editing. CV: Methodology, Writing – review & editing. EP: Software, Writing – review & editing. PT: Writing – review & editing. MA: Writing – review & editing. AM: Writing – review & editing. IN: Formal analysis, Methodology, Validation, Writing – review & editing. LO: Conceptualization, Funding acquisition, Writing – original draft, Writing – review & editing.
